# Blood pressure in Warmblood horses before and after a euglycemic-hyperinsulinemic clamp

**DOI:** 10.1186/1751-0147-57-S1-O5

**Published:** 2015-09-25

**Authors:** Katarina Nostell, Sanna Lindåse, Johan Bröjer

**Affiliations:** 1 Department of Clinical Sciences, Swedish University of Agricultural Sciences, Uppsala, Sweden

## Introduction

Insulin resistance (IR) in humans is related to hypertension and impaired vasodilation. Insulin administration has been shown to lower blood pressure both in insulin-resistant as well as in insulin-sensitive individuals.

## Objectives

The aim of the study was to investigate the association between insulin sensitivity and alterations in blood pressure in horses before and after a euglycemic-hyperinsulinemic clamp (EHC).

## Material and methods

A 3-hour EHC was performed in 13 Warmblood horses (11 mares, 2 geldings). All horses were clinically healthy but had variable degrees of insulin sensitivity, were some horses were defined as insulin resistant. Blood samples for analysis of plasma glucose and insulin were collected before the start of the EHC, every 10-min during the EHC and immediately after the EHC. Mean, systolic and diastolic blood pressure was measured before and after the EHC using an indirect oscillometric device. Insulin and glucose data from the EHC were used to calculate the mean rate of glucose disposal per unit of insulin during steady state (M/I-index).

## Results

Insulin administration decreased systolic, diastolic and mean arterial pressure in all horses. The M/I index was positively correlated with the decrease in systolic blood pressure (r^2^=0.55), but not diastolic and mean arterial pressure.

## Conclusions

This study indicates that horses with insulin resistance have a blunted response to the cardiovascular effects of insulin.

